# Assessing thermal imagery integration into object detection methods on air-based collection platforms

**DOI:** 10.1038/s41598-023-34791-8

**Published:** 2023-05-25

**Authors:** James E. Gallagher, Edward J. Oughton

**Affiliations:** 1 Geography and Geoinformation Science, George Mason University, Fairfax, VA 22030 USA; 2 Environmental Change Institute, University of Oxford, Oxford, Oxfordshire UK

**Keywords:** Computational science, Computer science, Scientific data

## Abstract

Object detection models commonly focus on utilizing the visible spectrum via Red–Green–Blue (RGB) imagery. Due to various limitations with this approach in low visibility settings, there is growing interest in fusing RGB with thermal Long Wave Infrared (LWIR) (7.5–13.5 µm) images to increase object detection performance. However, we still lack baseline performance metrics evaluating RGB, LWIR and RGB-LWIR fused object detection machine learning models, especially from air-based platforms. This study undertakes such an evaluation, finding that a blended RGB-LWIR model generally exhibits superior performance compared to independent RGB or LWIR approaches. For example, an RGB-LWIR blend only performs 1–5% behind the RGB approach in predictive power across various altitudes and periods of clear visibility. Yet, RGB fusion with a thermal signature overlay provides edge redundancy and edge emphasis, both which are vital in supporting edge detection machine learning algorithms (especially in low visibility environments). This approach has the ability to improve object detection performance for a range of use cases in industrial, consumer, government, and military applications. This research greatly contributes to the study of multispectral object detection by quantifying key factors affecting model performance from drone platforms (including distance, time-of-day and sensor type). Finally, this research additionally contributes a novel open labeled training dataset of 6300 images for RGB, LWIR, and RGB-LWIR fused imagery, collected from air-based platforms, enabling further multispectral machine-driven object detection research.

## Introduction

Despite the recent growth and proliferation of Machine Learning (ML) object detection algorithms, most approaches commonly focus on the visible light portion of the electromagnetic spectrum, for example, using Red–Green–Blue (RGB) images^1–4^. Hitherto, thermal Long Wave Infrared (LWIR) spectrum has received less research attention for ML object detection activities. While machine-assisted RGB models are effective during daytime periods, machine-assisted LWIR-models are generally more effective at night or during periods of decreased visibility^5–8^. Unlike RGB, LWIR provides superior edge enhancement of radiant object classes to further increase edge detection in object detection algorithms. Given the contrasting strengths and weaknesses between RGB and LWIR, a growing area of multispectral research examines the blending of these different capabilities with the ultimate aim of providing superior object detection^9–11^. For developing both RGB and LWIR models most software techniques are relatively similar. Although the pricing of LWIR sensors is becoming more economical, cost has traditionally been a prohibiting factor, limiting the amount of multispectral research activities taking place.

Another complementary technology that is rapidly proliferating and becoming easier to access is commercially available off-the-shelf drone platforms^12,13^. Increasingly, Uncrewed Aerial Systems (UASs) are being outfitted with not only RGB sensors to collect overhead imagery, but also with an array of other infrared-related sensors, such as LWIR (7.5–13.5 µm), to collect valuable multispectral data^14^.

Given these limitations, the literature currently lacks scientific evaluation metrics on how different thermal image fusion techniques affect model performance when utilizing object detection methods from drone platforms. This research therefore intends to investigate the following research question:

1. How do fused RGB-LWIR object detection models perform against separate RGB and LWIR approaches when measured at various fixed altitudes and different times of the day?

**Figure 1:**
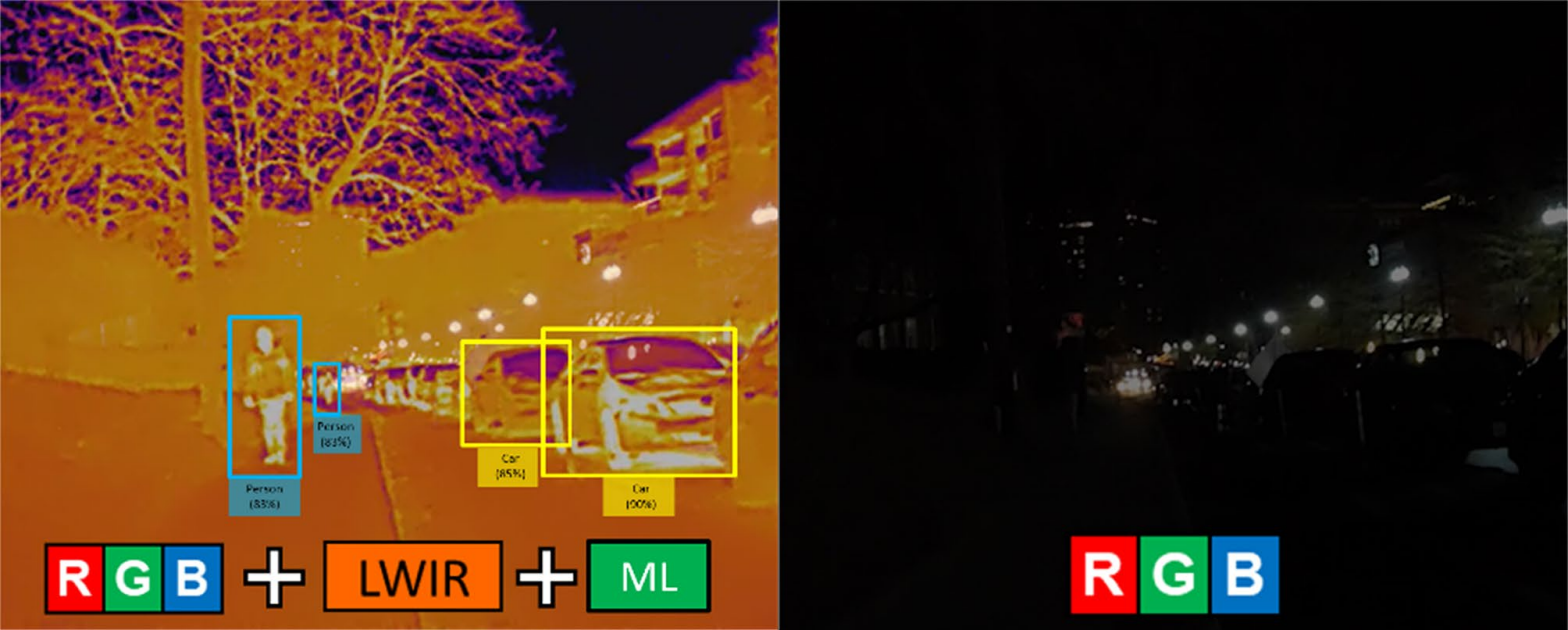
Comparative example of a nighttime scene with a blended RGB-LWIR approach on the left, and a traditional RGB image on the right.

The key contribution of this research is providing new quantitative scientific information for how RGB, LWIR, and RGB-LWIR object detection models perform from air-based drone platforms. The research focuses on identifying common object classes (cars, trucks etc.), as these provide generalizable insights for a wide range of object detection use cases in industrial, consumer, government, and military applications. Figure 1 illustrates the comparative differences between fused RGB-LWIR imagery versus traditional RGB imagery in a low visibility setting.

RGB-LWIR models deployed from UAS can be leveraged to solve a variety of spatial problems across diverse disciplines. An example use-case is the energy industry using RGB-LWIR object detection to assess pipeline integrity^15^. Deploying RGB and LWIR object detection on critical infrastructure can also increase operational efficiency, while also help prevent catastrophic failures^16^. Moreover, energy companies can use RGB-LWIR object detection to evaluate electricity transmission and distribution infrastructure performance^17^. Within transportation, RGB-LWIR object detection has also been used to monitor and rapidly identify faults in railway infrastructure^18^. Recently, RGB and LWIR air-based object detection can be applied to construction applications to detect harmful legacy substances, such as asbestos, as well as monitor home insulation efficiency^19,20^. Additionally, search and rescue teams can utilize UAS-deployed RGB-LWIR object detection to identify victims in any environment, regardless of ambient illumination and temperature^21,22^. The agriculture industry can also benefit by tracking and identifying specific livestock based not only on the thermal signature but also on the visible image of the animal^23^. RGB and LWIR object detection are also critical systems for both autonomous driving and advanced driver assistance systems^24^. Lastly, military and law enforcement entities can benefit by improving upon existing collection and surveillance capabilities^25,26^.

In the following section a literature review is undertaken. Then results are presented in Section "Results", before returning to the discussion to reevaluate the key research question in Section "Discussion". A method is described in Section "Methods" capable of answering the research question identified.

## Literature review

The existing literature identifies two key benefits for integrating LWIR with RGB to enhanced ML object detection models. Firstly, RGB sensors are limited in their capacity to detect in low visibility settings, or in situations where visibility is limited due to foliage, smoke or fog^27,28^. Therefore, integrating LWIR imagery enhances both human and machine three-dimensional (3D) depth perception when compared to traditional RGB imagery, providing an overall increase in situational awareness^29^.

Secondly, LWIR sensors are superior at segmenting the object of interest from the image background (‘edge detection’)^16^, provided that the object of interest is radiating a thermal signature (as illustrated visually already in Fig. 1). LWIR object detection is regularly adopted in military and homeland security use cases to detect illicit activity and identify targets, especially at night^30,31^. However, most infrared (IR) sensors for military and national security applications use near-infrared (NIR), which operates between 0.75 and 1.3 µm and does not work well for drone-based ML object detection models^32^.

In terms of the wider literature, one recent study evaluated ML object detection models that analyzed RGB and LWIR imagery to better identify humans from a ground-based system^30^. In adverse weather conditions, when attempting to identify humans, the LWIR model achieved a mean Average Precision (mAP) of 97.9% while the RGB model achieved a mAP of 19.6%^30^. Indeed, both LWIR and RGB models were tested, although no baseline performance metrics were provided for a blended RGB-LWIR approach. The research used ground-based sensors and utilized version 3 of the pre-trained convolutional neutral network ‘You Only Look Once’ (YOLOv3). A thermal dataset was used to attempt to identify humans and animals during various weather conditions ranging from clear conditions to inclement conditions with limited visibility. Although their LWIR model outperformed the RGB model, the performance gap was most significant when visibility was limited. The thermal ML model was also highly accurate in differentiating multiple object classes in a single image, reaching a recall of 98% with an F1 score of 97%^30^.

A separate research study recently used LWIR imagery to train an object detection model that achieved an average accuracy of 91.9% during periods of limited visibility^33^. However, it was identified that a shortfall of LWIR object detection is that LWIR cameras have difficulty identifying object classes at longer distance. As the object class is farther away, the thermal edges begin to blur and the thermal signature resolution deteriorates, making it difficult for the ML model to conduct edge detection^34^. Thus, because of this resolution decrease over distance, this supports the conjecture that fusing RGB with LWIR provides additional value in model performance.

Another research study that used LWIR sensors from a low-flying multirotor quadcopter collected thermal data to create a human detection model that identifies human heat signatures. The approach was applied to a rescue operations use case following natural disasters by using object segmentation and fusion technique called 4-channel^35^. The 4-channel ML model conducted “early fusion” of RGB-thermal images, performing better than the traditional “late fusion” model. This study focused on object segmentation of LWIR images taken from the UAS post-flight and did not conduct object detection from LWIR images or RGB-LWIR fused images.

The reliability of LWIR sensors to work in complex environments has led to adoption in numerous technologies. For example, LWIR sensors are used to advance semantic segmentation, classifying pixels in an image associated to a label class, with key use cases in autonomous driving^36–38^. However, a key issue in the application of this technology to autonomous driving is the low resolution and heavy noise present in LWIR images when compared to RGB methods^39^.

LWIR based object detection does present several key challenges for ML algorithms. One such issue is blurring in LWIR imagery caused by object movement or LWIR camera movement^40^. One study addressed this issue using a LWIR image restoration algorithm that conducts super-resolution reconstruction and deblurring while simultaneously running the object detection algorithm^40^. Although the methods to deblur LWIR images does increase the overall accuracy of the object detection results, it also requires increased computer processing to conduct simultaneous image restoration and object detection when conducting real-time inference on edge devices. In this research study there is an undetermined level of image blurring induced by the moving airframe with RGB-LWIR cameras.

Another issue with LWIR object detection is that there exists a shortage of publicly available LWIR datasets or pre-trained LWIR models^41^. Indeed, there are multiple pre-trained RGB ML models and datasets to choose from, but very few LWIR datasets and pre-trained models. Labeled LWIR datasets are scarce because they are expensive to collect and produce, and LWIR cameras are not widely available to the same degree as RGB cameras^41,42^.

A key benefit to blended RGB-LWIR is the ability to adjust fusion levels between the RGB-LWIR sensors as ambient and ground temperatures increase, creating an effect called thermal crossover. When the target object is the same temperature as the ground, thermal cross over takes place leading to a loss of contrast between the target object and the ground^43^. Depending on the environment and season, thermal crossover typically occurs twice a day. Via a ground based LWIR ML object detection model approach, thermal crossover is not as large an issue because the horizon provides a dark background to contrast against thermal target objects. However, from a UAS the bird’s-eye view of the ground offers significantly lower contrast with the target object. When using an LWIR camera without an RGB camera or having the ability to conduct RGB-LWIR fusion, the ambient and ground temperature must be factored in prior to flight.

Thermal object detection is also advantageous because of the ability to conform an image to a desired color palette^44^, thereby reducing the overall number of colors compared to RGB images^45^. Often, RGB images can have backgrounds that blend in with the object of interest^46^, making object detection a more challenging task. In contrast, thermal imagery highlights the object of interest and provides a consistent color palette^47^. The study results will now be presented.

## Results

The mAP results are reported for RGB, LWIR and RGB-LWIR models at various fixed elevations to measure performance changes, as well as daily time periods. Therefore, the findings are segmented for eight elevations, including 15 m (50 ft), 30 m (100 ft), 45 m (150 ft), 61 m (200 ft), 76 m (250 ft), 91 m (300 ft), 106 m (350 ft), and 121 m (400 ft). The test area selected was a busy four-way intersection in Gaithersburg, Maryland. This intersection was selected because of the complex environmental blend of objects among various lighting shades. The collection site also provided multiple vantage points of vehicles entering and leaving the intersection, thus helping to generate realistic data.

The best overall predictive performance was exhibited by the RGB-LWIR model (with a mean mAP of 59.8%), followed by the traditional RGB model (58.6%). In contrast, the LWIR model performed the poorest (with a mean mAP of 36.3%). The best individual performing instance was the blended RGB-LWIR hybrid at 47 m elevation during the Pre-Sunrise period (with a mean mAP of 94.6%). Moreover, the worst performing instance was the LWIR model at 125 m during the Post-Sunrise period (with a mean mAP of 2.1%).

**Figure 2:**
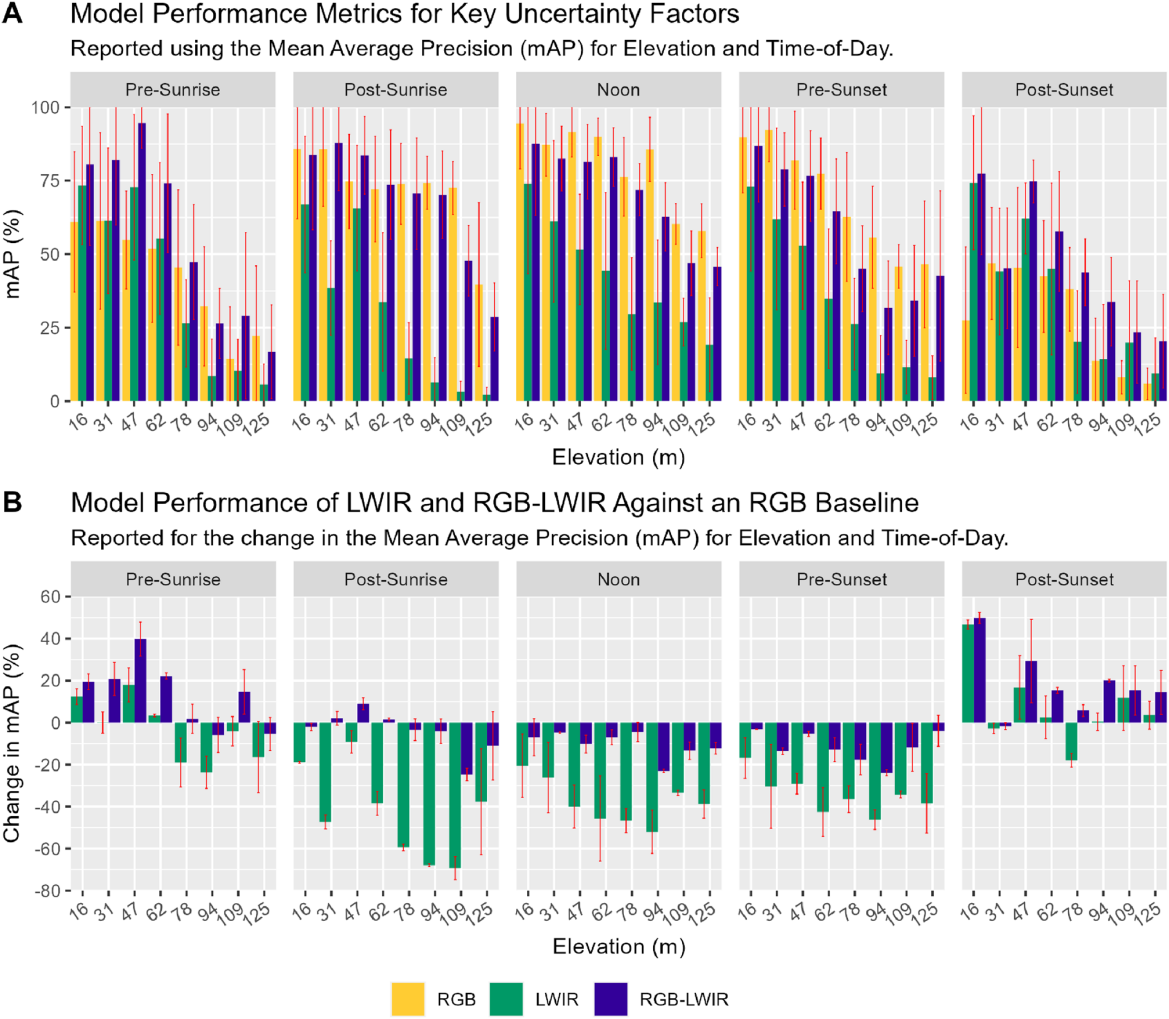
Panel plot of model performance metrics for key uncertainty factors. Conference intervals reported at 1 standard deviation.

Figure 2A graphically depicts all 120 model performance data points for each model type, elevation, and time-of-day period. The RGB-LWIR model performed very strongly during periods of limited visibility (Pre-Sunrise and Post-Sunset), while the RGB models exhibited superior performance during daytime periods of visibility. In particular, the RGB-LWIR fusion approach demonstrated strong predictive power during the Pre-Sunrise and Post-Sunset periods between elevations of 16 m and 67 m. During periods of clear visibility, RGB and RGB-LWIR mAP decreases gradually as elevation increases. Conversely, during periods of limited visibility mAP model performance decreases at a quicker rate, with performance declining upwards of 78 m. Although largely inferior in performance when compared to the other models, the LWIR performance was generally consistent across all five illumination periods.

As visualized within Fig. 2B, when using the traditional RGB model as a baseline, the RGB-LWIR model had up to a 49.9% increase in performance during the Post-Sunset period. Out of the eighty total elevation and time-of-day data points, the RGB-LWIR approach ranked in all top fifteen places with mean mAP values averaging 82.7%. In contrast, while the LWIR model achieved the bottom twelve lowest ranking positions with mean performance averaging 8.6%. The RGB-LWIR model performed best overall at 47 m during Pre-Sunrise hours (with a mean mAP of 94.6%) and performed worst overall at 121 m, also at Pre-Sunrise hours (with a mean mAP of 16.7%).

The RGB approach achieved the highest mAP during periods of clear visibility (Post-Sunrise to Pre-Sunset). Figure 2B visualizes model performance against the RGB baseline, demonstrating that RGB approaches are best suited for daytime conditions while the RGB-LWIR approach is best suited for nighttime conditions. The greatest difference between the RGB and RGB-LWIR model performance during clear visibility conditions was at Noon (7.25% difference in mean mAP), followed by Post-Sunrise (3.2% difference in mean mAP) and then Pre-Sunset (1.2% difference in mean mAP). The RGB model performed best at 16 m at Noon (94.5% in mean mAP) and performed worst at 125 m during Post-Sunset hours (5.8% in mean mAP).

The LWIR approach had the lowest predictive power of all three models, with a negative performance change of up to − 69.2% when compared to the RGB model baseline. The three least performing instances for LWIR occurred at the Post-Sunrise period with negative performance values ranging between − 59.0% and 69.2%. Noon was the next lowest performing period for LWIR, with the top 3 negative performance values reaching RGB baseline differences between − 52.03% and 39.95%. The LWIR model also suffered the sharpest decrease in performance over elevation, with the worst performance localized between 94 and 121 m. The LWIR model performed best at 16 m during the Post-Sunset period (74.3% mAP) and performed worst at the Pre-Sunset period at 94 m (9.5% mAP).

During Post-Sunrise, RGB and RGB-LWIR approaches both performed similarly below 94 m, with RGB-LWIR performing consistently between − 4% and 8% of the RGB baseline. LWIR regularly performed far below the RGB baseline, ranging between − 9% and − 69.3%, explained by factors already well identified in the literature (e.g., increases in distance lead to decreased resolution when compared to RGB). Both LWIR and RGB-LWIR performance deteriorated rapidly at 109 m and 125 m when compared to the RGB baseline (for example, between − 11% to − 69.3% below the traditional RGB approach). The LWIR model performed the worst during periods of clear visibility, for example, with the worse LWIR performance occurring Post-Sunrise (− 24.7% from RGB baseline), Pre-Sunset (− 12.1% from RGB baseline) and Noon (− 11.3% from RGB baseline).

**Figure 3:**
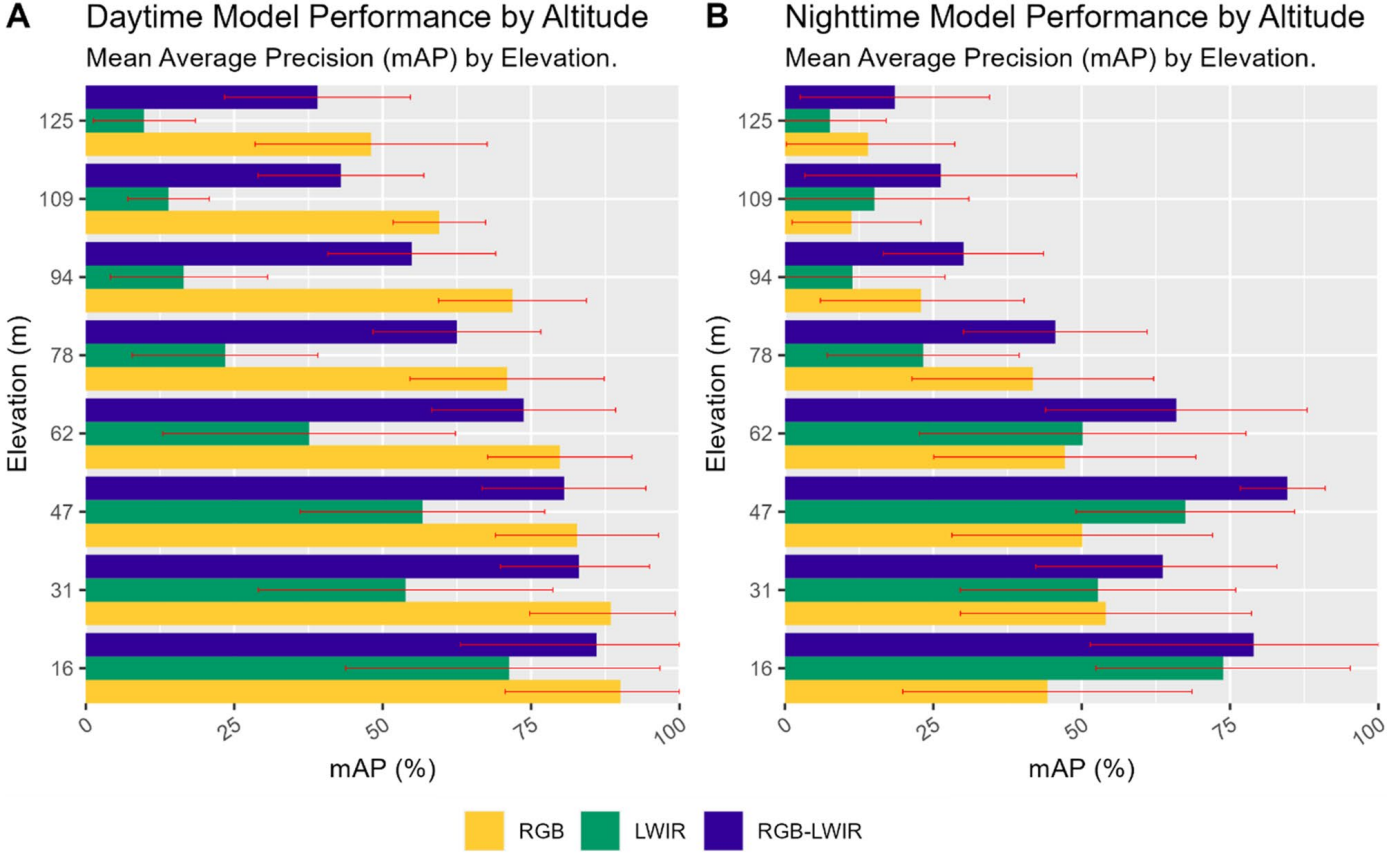
Model performance during night-day periods. Confidence intervals reported at 1 standard deviation.

In Fig. 3A, when analyzing model performance by elevation and daytime periods (Post-Sunrise, Noon, Pre-Sunset) both RGB and RGB-LWIR models performed similarly at all elevations. Both models had near identical mAP performance between 16 and 62 m. Both RGB and RGB-LWIR models also shared comparable mAP performance decreases over different elevations. Both RGB and RGB-LWIR models achieved the highest mAP at the lowest altitudes and gradually decreased mAP performance over vertical distance, losing approximately 1–5% in mAP performance every 15 m.

In contrast, in Fig. 3B when analyzing model performance at night, the RGB-LWIR model significantly outperformed both RGB and LWIR approaches. Unlike the RGB model which had a consistent reduction in mAP over distance, the RGB-LWIR model performed consistently between 16 and 47 m with performance slightly increasing over increasing altitudes (14.1% mAP increase between 16 and 47 m). At 47 m, the RGB-LWIR approach had a higher mAP (94.6%) than the RGB model, with the best predictive performance at the same altitude during periods of daytime illumination (91.5%).

## Discussion

Given the lack of baseline performance metrics evaluating RGB, LWIR, and RGB-LWIR object detection machine learning models, especially from air-based platforms, this study undertook such an assessment. Whereas most object detection models have commonly focus on utilizing the visible spectrum using RGB imagery, the method undertaken here fused RGB with thermal LWIR (7.5–13.5 µm) images.

Thus, over 6300 training images were collected for RGB and LWIR sensors, mounted on a multirotor drone, creating an openly available fused RGB-LWIR dataset. Three object detection models were then trained, each based on one of the three image types identified (RGB, LWIR and RGB-LWIR). After training, an additional 1200 testing images were collected from eight separate altitudes at five separate periods of the day. These images were then used to assess mAP performance for key uncertainty factors (altitude and time-of-day).

This discussion will return to the research question identified earlier in this paper, to discuss key findings, now that results have been obtained and reported.

*How do fused RGB-LWIR object detection models perform against separate RGB and LWIR approaches, when measured at various fixed altitudes and different times of the day?*

When analyzing the mean average across all mAP results, the RGB-LWIR method outperformed the RGB approach by 5.6%. Although the mean mAP is similar between these two models, both performed inversely under different illumination conditions and altitudes. For example, the RGB-LWIR approach was superior for conducting object detection in periods of limited visibility. This finding is counterintuitive to the belief that LWIR by itself would be the best suited sensor to conduct object detection in nighttime settings. The RGB-LWIR fusion helped to dampen long-distance blurring and thus the resolution loss that LWIR sensors suffer from as object classes become farther away. The RGB fusion allows for an additional edge to be overlayed on the thermal signature of the object class, providing edge redundancy and edge emphasis, both which are vital in supporting edge detection machine learning algorithms. The LWIR fusion with RGB was only beneficial if the object classes were radiating thermal energy between 7.5 and 13.5 µm. Cold object classes would not be detected by the LWIR model and would thus be reliant on the RGB model for detection. The novelty of the RGB-LWIR model is that it combines critical edge information from objects with both visible-RGB edges and non-visible radiant-specific edges to increase performance as well as model resiliency. Examples of radiant-specific edges can be vehicle wheels, engine compartments, exhaust systems, and people.

Counter to expectation, the LWIR model performed best during the Post-Sunset period. Surfaces during Post-Sunset periods generally retain ample amounts of heat from the day. Increased ground surface temperature provides less contrast to the object class (thermal crossover) which would reduce edge detection. The Post-Sunrise period is associated with cooler ground temperatures, thus providing greater contrast to warm object classes, and resulting in higher predictive power. Post-Sunset ground temperature is one of the warmest daily periods, decreasing the background contrast of object classes. The LWIR model had the best performance in Post-Sunset conditions, but performed very poorly in Pre-Sunrise conditions. One limitation is that these findings may be season-dependent, and therefore further research should be conducted during a greater annual range of months (particularly summer months) to further quantify these differences in sensor performance during larger temperature ranges.

When visualizing mAP metrics across different periods of the day, there was a slight upward trend in the Pre-Sunset results between 109 m and 121 m. This upward trend may be due to variety in image quality due to atmospheric disturbances. For example, the images tested at 121 m may be of higher quality than the images at 109 m due to drone stablility, image angle and lighting angle. Sun position (sunrise and sunset) may have also played a role in RGB sensor and model performance. For a truly consistent experiment, a static object class can be used in future research to measure model performance and sensor type over elevation and illumination levels. However, this approach is not necessarily feasible for realistic applications where complex scenes with changing or moving object-classes are present.

When analyzing model performance over elevation, during daytime hours, model performance decreased gradually over elevation. Excluding LWIR, performance generally decreased consistently between 1–5% over every 15 m, as reported in Fig. 3A. During nighttime hours the decrease in mAP was much sharper, with performance dropping significantly at 62 m (15.3% reduction in mAP).

The model performance metrics from this research indicate both future research opportunities and research limitations in deploying air-based multispectral object detection models. For example, the results demonstrate that not one specific object detection model type is best suited for all conditions, and that each ML model type has its own strengths and weaknesses for certain situations. More specifically, the RGB model performed best during daytime hours due to superior resolution across all altitudes. In contrast, the RGB-LWIR model performed best at night because of superior edge refining characteristics. However, the LWIR model exhibited the lower performance in all daily time periods because of rapid resolution deterioration as elevation increased.

To conclude, this research successfully quantified the performance of three unique models and found that the RGB-LWIR model generally performed the best. This is because RGB-LWIR provided consistent detection performance across many daily time periods with heterogenous illumination levels. Indeed, the blended RGB-LWIR approach only performed 1–5% behind the RGB approach at various altitudes during periods of clear visibility, while also having the advantage of operating in poor visibility settings. One final benefit is the open dataset generated from this research. Thus, this labeled imagery could be integrated as training data into future air-based LWIR multispectral object detection research. Lastly, two key contributions are made from this research of high relevance to the scientific community. Firstly, the factors affecting model performance from drone platforms are quantified (including distance, time-of-day and sensor type), which are highly relevant to the development of new multispectral image recognition algorithms and future use cases/applications. Secondly, this research generated the first air-based multispectral training dataset of labeled data consisting of 6300 images. Other researchers can therefore utilize this resource for training new multispectral models (with the production of this dataset constituting two full months of labeling work alone).

## Methods

This method describes key steps including sensor selection, data collection, image processing and labeling, model training and the testing of air-based models. When these method steps are combined, they produce a final set of model performance metrics capable of answering the research question identified for investigation.

### Sensor selection

The LWIR camera selected for this research is the FLIR (Forward-Looking Infrared) Vue Pro R. The FLIR Vue Pro R is a radiometric capable camera designed specifically for drones and costs $2914 USD. The field of view (FOV) for the camera is 45° with a lens diameter of 6.8 mm^48^. The 30 Hz variant of the FLIR Vue Pro R will be used. Although the 30 Hz FLIR Vue Pro results in a higher frames per second rate (30 FPS) compared to the 9 Hz variant (9 FPS), the 30 Hz is export controlled and cannot be purchased outside of the United States. Both 30 Hz and 9 Hz variants produce the same LWIR resolution. The camera resolution is 336 × 256 pixels and has a spectral band of 7.5–13.5 µm. The operating temperature range for the FLIR Vue Pro R is – 20 °C (− 4 °F) to 50 °C (122 °F)^48^.

The RGB camera selected for this research is the RunCam 5 Orange, which is designed for drone applications and costs $110 USD. The RunCam 5 uses a Sony IMX377 12 megapixel image sensor which has a FOV of 145° with adjustable resolution, ranging from 1080P at 60 FPS to 4 K at 30 FPS^49^. 1080P (1920 × 1080 pixel resolution) at 60 FPS (60 Hz) will be used for this research. Shutter speed, ISO, color style, saturation, exposure, contrast, sharpness and white balance are all set to the default settings.

### Data collection

**Figure 4:**
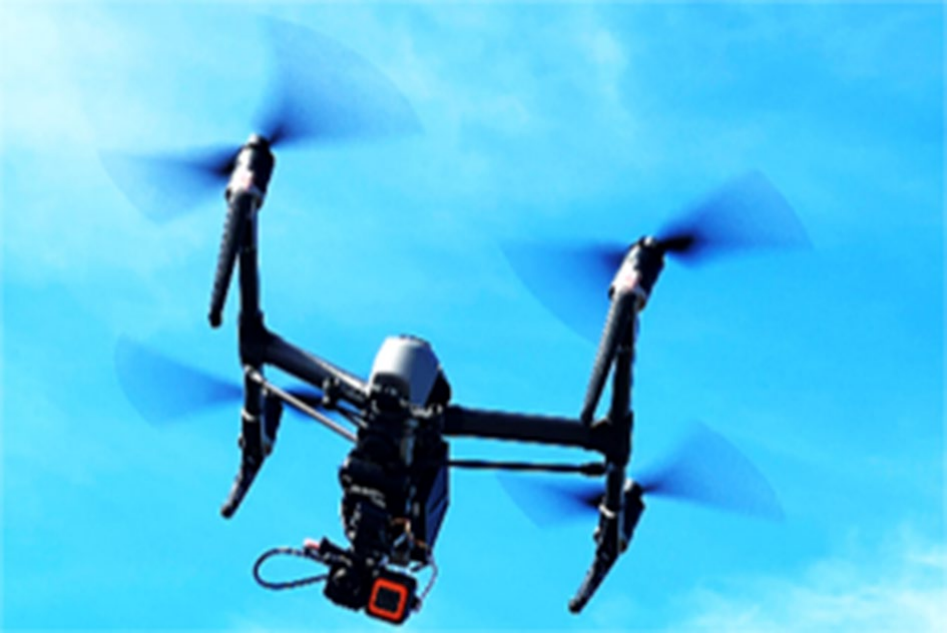
The primary air-based platform used for this research (the DJI Inspire 2) carrying the RGB-LWIR payload.

Overhead imagery collection for the air-based ML models is collected from the DJI Inspire 2 (Fig. 4). The RGB and LWIR cameras on the multirotor are co-aligned to maintain the same field of view to ensure that similar images are being collected between the two sensors^50^. Data are collected during various times of the day at different temperatures to ensure data diversity. Footage is recorded and extracted on the camera’s micro-SD cards. Frames of interest from the footage are then extracted and converted into images to train the ML model. Images are also collected from various altitudes to ensure image diversity and to help reduce model performance loss at higher altitudes.

A 3D printed component for the RGB camera was designed and printed to be able to directly mount the RGB camera to the LWIR camera. The 3D printed mounting bracket reduces parallax as well as ensures the same FOV of both cameras. This fixed FOV makes fusing the LWIR and RGB footage easier in Adobe Premier Pro. The file to print the mounting bracket can be found in the data availability section.

**Figure 5:**
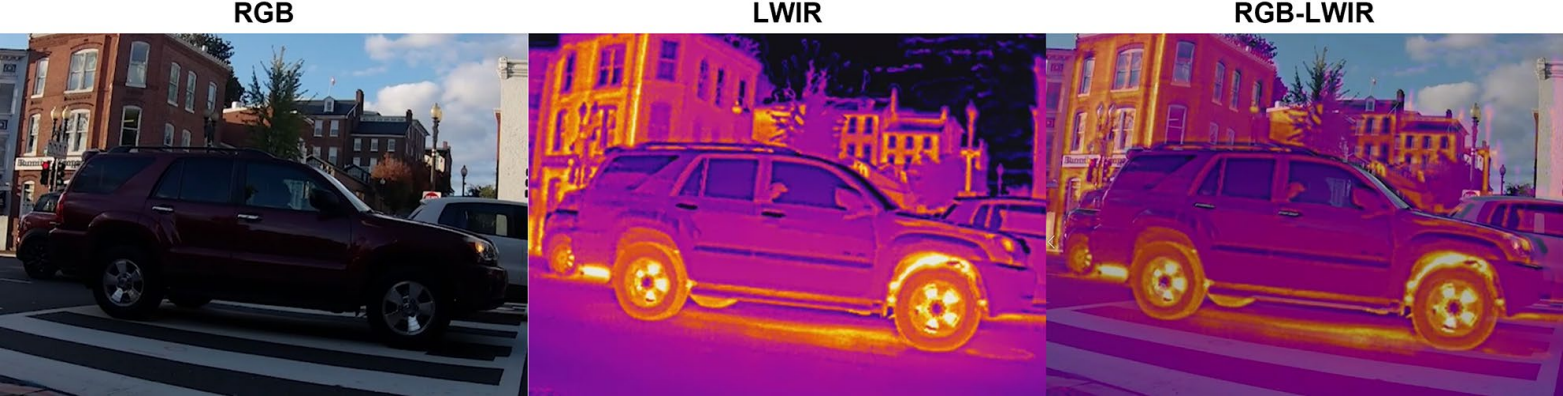
An example of an RGB image (left), an LWIR image (middle) and a RGB-LWIR fused image (right).

The original training images are collected from various camera angles at five different times of the day^51^. These original images consist of 100 RGB and 100 LWIR extracted from the full-motion video footage with each object class. The RGB and LWIR footage is then fused in Adobe Premier Pro with a 50–50 fusion ratio to create an additional 100 images for the fused RGB-LWIR dataset (Fig. 5). Geometric distortions (skew) were not addressed. Photometric distortions (image degradation from Moiré pattern noise) were addressed by adjusting the RGB layer during the fusion process to prevent double edges produced by parallax from the two sensors. As distance and parallax from the target object increased, the RGB layer was adjusted and scaled, ensuring a consistent clean overlap between RGB and LWIR footage.

### Image processing and labeling

**Figure 6:**
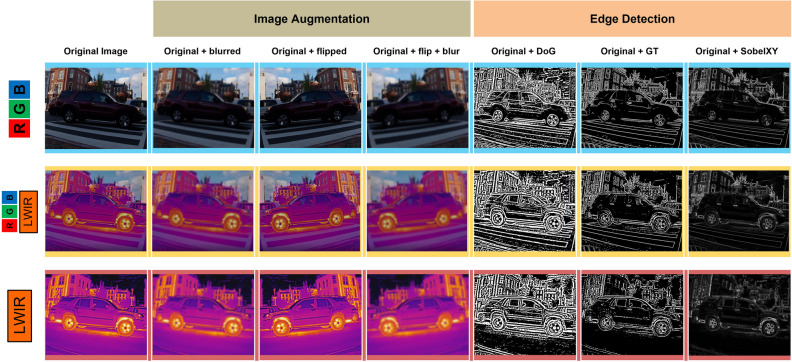
Visual examples of the six image processing techniques applied.

Image Processing (IP) techniques are then applied to the original images to increase the quantity available in the training dataset, while simultaneously generating edge-enhanced images to increase model performance^52,53^. Six image augmentation and edge detection techniques are carried out to help increase model performance which include flipping, blurring, blurring & flipping, Gaussian Thresholding (GT), Difference of Gaussians (DoG) and Sobel-XY^54^. See Fig. 6 for a visual example of each of these techniques for RGB, LWIR and fused RGB-LWIR. The blurred and blurred + flipped image augmentation techniques are especially useful because of video vibrations caused by the oscillatory motions from the airframe’s propellers^55^. Model training on blurred images helps to ensure that the model will continue to work when frames are blurred due to camera movement, target object movement, or both. Although counterintuitive, training ML models with blurred images tends to increase detection rates and confidence levels^56^. All code for generating and exporting augmented images can be found in the image processing link in the data availability section^57^.

After image processing, a total of 5400 new training images are generated, resulting in a total of 6300 total images. 90% of the dataset (5670 images) is used for training, 5% (315 images) is used for validation, and the remaining 5% (315 images) is used for testing. None of the newly generated images are used for testing. This is to ensure that testing results are similar across all ML models. Lastly, all images are labeled using LabelImg, which is an open-source python based image labeler^58^.

### Model training

This research utilizes YOLOv7 as the Convolutional Neural Network (CNN) to perform object detection^59^. YOLOv7 was selected because to date it surpasses all existing object detectors in terms of speed and accuracy^60^. YOLOv7 is considered one the fastest open-source object-detection models currently available^60–62^. A primary shortfall of this family of object detection models is that YOLO approaches can struggle to detect smaller objects within an image, which is primarily due to spatial constraints in the algorithm^63,64^. There are six YOLOv7 models currently available. The standard YOLOv7 variant is used for this research study^65^.

The standard YOLOv7 model is the smallest in size, easy to deploy in the field on edge devices, and also the fastest model (2.8 ms average inference time)^66^. YOLOv7-E6E is the largest model, attaining on average 4.7% higher mAP than the standard YOLOv7 model used in this research. However, it is also 16.9 ms slower on inference than the standard model. A comparative analysis of three other YOLO models was conducted to assess how different pre-trained neural networks performed when presented with the same RGB, LWIR and RGB-LWIR labeled training dataset. The three object detection models assessed were YOLOv5, YOLOv7E6E, and YOLOv8. All of these YOLO models use PyTorch as their deep learning framework. YOLOv8 is the newest variant of the YOLO family and was released as this research was culminating near completion.

**Figure 7:**
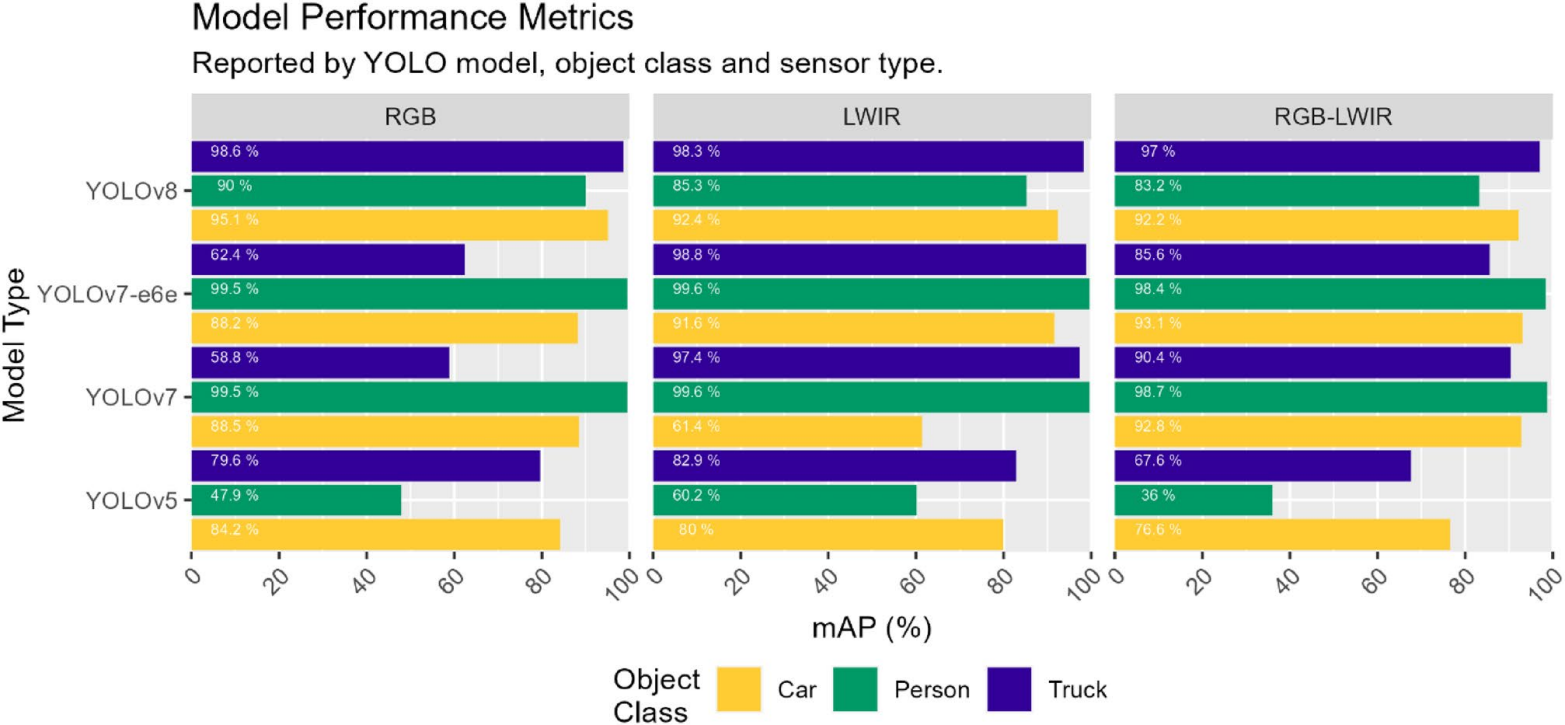
Model, object class and sensor performance when presented to different pre-trained object detection models.

Figure 7 depicts the mean average performance of the three sensor types as they relate to their respective object detection model type along the y-axis. The mean object class mAP is visualized along the x-axis to demonstrate which model types performed best at identifying certain object classes. YOLOv8 outperformed other models in identifying larger object classes (car, truck), but had difficulty in identifying smaller object classes (person). The YOLOv7 and YOLOv7E6E models performed exceptionally well in identifying people. YOLOv5 performed the poorest and had the most difficulty in identifying people. Conversely, sensor performance was dependent on the type of object-detection model selected. The RGB model performed the best in YOLOv8 (95.5% mAP) but in contrast performed the worst in YOLOv7E6E (83.3% mAP). LWIR had a significant increase in mAP performance between YOLOv7 and YOLOv7E6E (10.5% increase). The RGB-LWIR models performed generally consistently between YOLOv7, YOLOv7E6E and YOLOv8. YOLOv5 overall performed the worst, falling 24.2% mAP behind YOLOv8.

Using YOLOv7 three models were trained: the RGB, LWIR and RGB-LWIR models. The RGB and LWIR models were selected because of the common use of these sensors in research today, as well as to establish benchmark metrics that could be used to better quantify RGB-LWIR model performance. The RGB-LWIR model is trained on images with an equal part fusion of 50% RGB and 50% LWIR images. Although the fusion ratio can be adjusted to optimize model performance based on ambient temperature and illumination levels, the RGB-LWIR model was trained on equally fused images to standardize results. Each model was trained on 300 original unprocessed images and 1800 images generated from image processing, resulting in a total of 2100 images used to train each model. The labeled image dataset used to train each model was equally divided by the three object classes of car, truck, and person, resulting in 700 images total for each object class. The model is trained through 55 epochs. This number was selected to prevent overtraining. There is an imbalance in the number of car and truck labels in the dataset, making overfitting a possibility if the models are trained through too many epochs^67^. Cars have the most labels in the dataset while trucks have the least. Training the dataset beyond the 55 epochs selected may result in an increase in false positives, thus decreasing the mAP of the model. After the completion of training the three models (RGB, LWIR and RGB-LWIR) are ready for evaluation from drone-based imagery at different periods of day at various altitudes.

### Testing air-based models

**Figure 8:**
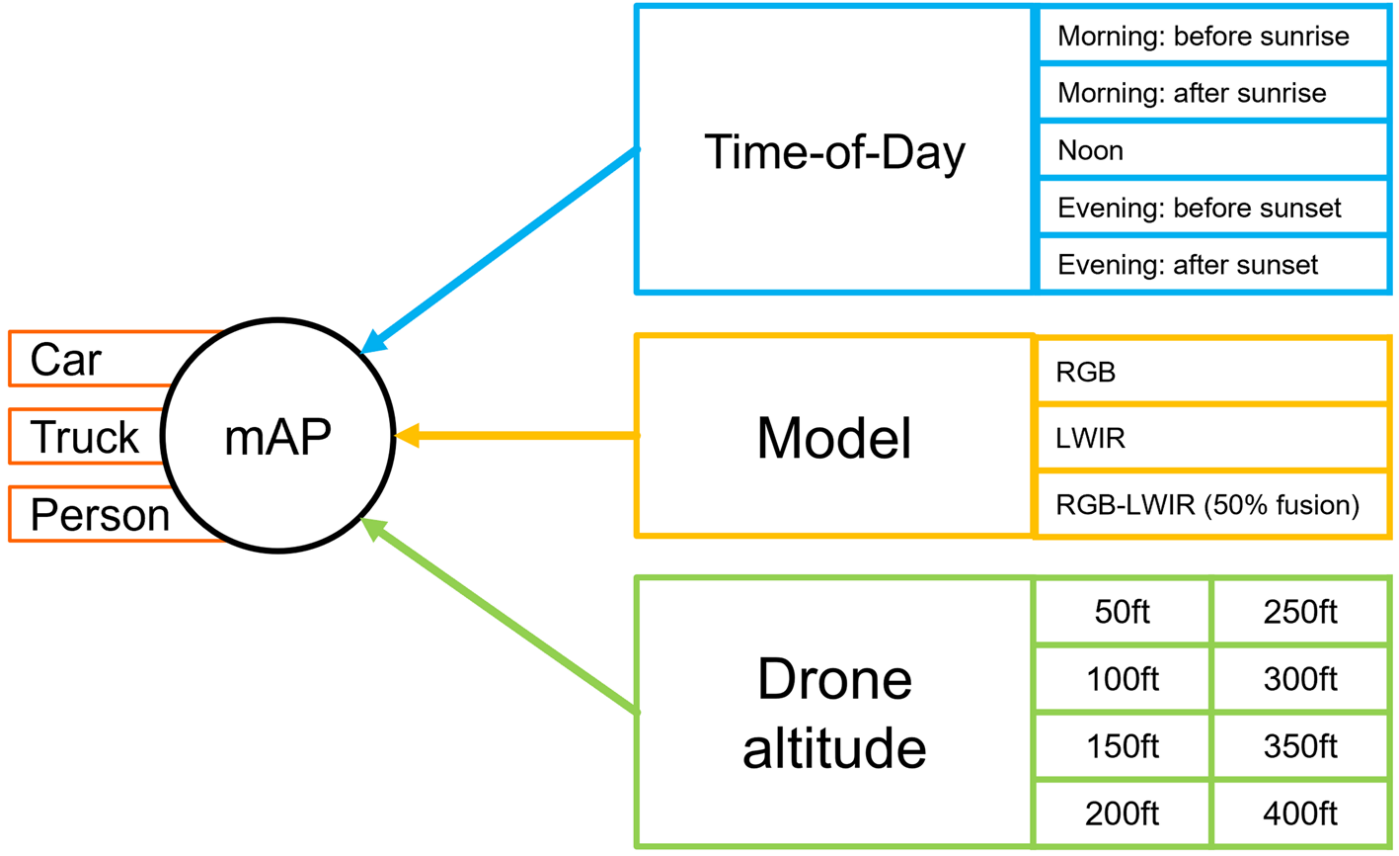
The research approach given key uncertainty factors, including time-of-day, image model and altitude.

A multirotor drone is utilized to fly at fixed elevations to determine inference performance via mAP for both sensors and all three model types. As indicated in Fig. 8, to assess the models and sensors new test images will be extracted from video footage, separate from those used for training, collected at 15 m (50 ft), 30 m (100 ft), 45 m (150 ft), 61 m (200 ft), 76 m (250 ft), 91 m (300 ft), 106 m (350 ft), and 121 m (400 ft). Footage cannot be collected above 121 m due to Federal Aviation Administration (FAA) drone regulation that prohibit drones from flying above 121 m (400 ft). Additionally, data will be collected at five different periods of the day. These include Pre-Sunrise (low-thermal cross-over, low illumination), Post-Sunrise (low-thermal cross-over, medium illumination), Noon (high-thermal cross-over, high illumination), Pre-Sunset (high-thermal cross-over, medium illumination) and Post-Sunset (high-thermal cross-over, low illumination). Atmospheric and location related metadata will also be recorded prior to each flight, to support both this study but also the reusability of images in future research. This metadata includes temperature (C°), wind speed (meters per second), illumination (lux), time, date, and location.

Five test images will be extracted at every elevation for each image type. This will result in 120 images (5 RGB, LWIR and RGB-LWIR images across the 8 elevations) per flight, with 600 labeled images (5 flights) per daily period. Following ten full flights, a total of 1200 test images is collected to evaluate model and sensor performance. When calculating mAP for test images, variables will be constrained to a confidence level of 10% with an intersection of union (IoU) of 65%. After executing the test code, the notebook exports critical metrics such as precision, recall, precision-recall curve, mAP@.5 and mAP@.5:95. For this research, only mAP@.5 will be used to measure sensor and model performance at fixed elevations. The labeled test image dataset and test script can be found in the Test Data link and YOLOv7 Training Code notebook link in the data availability section (Supplementary Information).

## Data Availability

The datasets generated during and analyzed during the current study are available in the Zenodo repository. Links to the code and datasets to the Zenodo repositories are provided in the below hyperlinked text. Air-based labeled data for all object classes: https://zenodo.org/record/7465521#.Y6Jk0XbMJD8, air-based ML model weights: https://zenodo.org/record/7466077#.Y6KiEXbMJD8, image processing code: https://github.com/jmansub4/RGB-LWIR_YOLOv7_training_testing, YOLOv7 training & testing code: https://github.com/jmansub4/RGB-LWIR_YOLOv7_training_testing, inference videos: https://zenodo.org/record/7469011#.Y6M04HbMJD8, test images with labels (images at elevation with labels): https://zenodo.org/record/7591134#.Y9lhx3bMJD8, 3D printed RGB mount: https://zenodo.org/record/7460106.
